# Antimicrobial resistance trends in fecal *Salmonella* isolates from northern California dairy cattle admitted to a veterinary teaching hospital, 2002-2016

**DOI:** 10.1371/journal.pone.0199928

**Published:** 2018-06-28

**Authors:** Kelly E. Davidson, Barbara A. Byrne, Alda F. A. Pires, K. Gary Magdesian, Richard V. Pereira

**Affiliations:** 1 Department of Population Health and Reproduction, School of Veterinary Medicine, University of California, Davis, United States of America; 2 Department of Pathology, Microbiology & Immunology, School of Veterinary Medicine, University of California, Davis, United States of America; 3 Department of Medicine & Epidemiology, School of Veterinary Medicine, University of California, Davis, United States of America; Universita degli Studi di Napoli Federico II, ITALY

## Abstract

Nontyphoidal *Salmonella* infections contribute to approximately 1.2 million annual illnesses in the United States. Historical and recent outbreaks have been associated with dairy products, ground beef, and direct contact with cattle. *Salmonella* antimicrobial resistance (AMR) is a serious concern that can reduce successful treatment of infections, increasing recovery time, medical costs, and mortality rates in humans and animals. This highlights the need to track AMR in *Salmonella* isolated from cattle to improve treatment plans, manage trends in AMR, and prevent future AMR development. A total of 242 *Salmonella* isolates were retrieved from 9,162 cattle fecal samples submitted to the University of California, Davis Veterinary Medical Teaching Hospital from 2002 to 2016. These isolates were tested for antimicrobial susceptibility using a standardized broth dilution panel. Multidrug resistance (MDR) to three or more classes of antimicrobials was observed in 50.8% of isolates, and the most common MDR pattern was amoxicillin-ampicillin-cefoxitin-ceftiofur-ceftriaxone-chloramphenicol-streptomycin-tetracycline (23.2%). There were significantly greater odds for antimicrobial resistance to aminoglycosides (OR: 2.03, *95% CI*: 1.1–3.7*)*, penicillins (OR: 1.87, *95% CI*: 1.007–3.5), and tetracyclines (OR: 1.87, *95% CI*: 1.017–3.4) for the 2002–2009 period when compared to the 2010–2016 period. The most prevalent MDR serotypes were Newport (100% MDR, n = 52), Typhimurium (100%, n = 20), and Dublin (71% MDR, n = 46). Risk factors associated with higher odds for isolating MDR *Salmonella* included isolates from calves when compared to adult cattle (OR: 22.0; *95% C*.*I*.: 3.9–125.7), and isolates obtained from cattle suspect of having salmonellosis versus from the infectious disease control surveillance program (OR:13.7; *95%C*.*I*.: 2.8–66.8). Despite a temporal trend for reduced AMR to most antimicrobial drug classes, a lack of this observed in the 2002–2009 period when compared to the 2010–2016 period for important drug classes such as cephalosporins (OR: 1.6, *95% CI*: 0.87–3.1*)*, and a trend for temporal increase in resistant to quinolones drugs (*P* value 0.004) highlight the relevance of AMR surveillance in cattle with *Salmonella* infections with the aim of targeting future prophylactic interventions.

## Introduction

Nontyphoidal *Salmonella* is categorized by the Centers for Disease Control and Prevention (CDC) as one of the top eighteen drug-resistant threats in the United States, associated with an estimated 1.2 million infections, 100,000 antimicrobial-resistant infections, and 300 million dollars in medical costs annually [1]. Though nontyphoidal *Salmonella* infections are rarely life-threatening in healthy individuals, the presence of antimicrobial resistance (AMR) in this organism can complicate and impede patient recovery and imposes an even greater risk for at high risk populations (e.g. immunocompromised, children and elderly) [2]. Humans can become diseased with nontyphoidal *Salmonella* via direct or indirect contact with infected livestock, which are in most cases asymptomatic. Some examples of disease transmission include: direct contact with an infected animal, consumption of produce contaminated by manure from infected animals, or consumption of beef, chicken, or other animal products derived from infected animal [3].

Specifically, dairy cattle and dairy products have been demonstrated to be potential reservoirs for nontyphoidal *Salmonella* including serotypes known to display AMR and cause foodborne illness in humans, such as *Salmonella* serotypes Newport and Typhimurium [4]. A recent, 2015–2016 outbreak affecting 36 people in ten states was linked to *Salmonella* serotype Heidelberg in dairy calves, and isolates of these organisms were found to be multi-drug resistant (MDR) [5]. Recent *Salmonella* outbreaks and observed resistance to medically-important drugs such as cephalosporins [6], highlight the continuing need for spatio-temporal quantification of AMR in non-typhoidal *Salmonella* in dairy cattle. This will facilitate estimating areas with higher risk for outbreaks caused by drug-resistant strains, facilitating prevention and management to reduce the spread and dissemination of AMR *Salmonella*.

Judicious use of antimicrobials is increasingly emphasized in the scientific and medical communities with major efforts directed toward reducing and refining use of drugs in livestock production, as this constitutes a large portion of overall antimicrobial use [7]. Recently, policymakers demonstrated high prioritization of antimicrobial stewardship in the livestock industry through the Veterinary Feed Directive (VFD) effective in 2015 [8] as well as the California Senate Bill 27 (SB27), which will come into effect in January 2018 [9]. The aim of the SB27 is to improve veterinary oversight of antimicrobial drugs in livestock and promote collaboration between veterinarians, producers, research scientists, and the government to better utilization of medically-relevant antimicrobials, increasing research on AMR bacteria and improving drug use in livestock management. To better understand spread and dissemination of antimicrobial resistance, this study aims to describe antimicrobial resistance trends in *Salmonella* isolated from dairy cattle in northern California.

The objective of this study was to identify trends in AMR of *Salmonella* isolates obtained from cattle fecal samples isolated and tested in the University of California, Davis William R. Pritchard Veterinary Medical Teaching Hospital (VMTH) microbiology laboratory between January 1, 2002 and December 31, 2016. These data provide initial findings on antimicrobial resistance of *Salmonella* overtime for area veterinarians and stakeholders and supporting information to manage antimicrobial use and more finely target research efforts to improve animal health and food safety.

## Material and methods

### Study design

All *Salmonella* isolates obtained from dairy cattle fecal samples submitted to the University of California, Davis William R. Pritchard Veterinary Medical Teaching Hospital (VMTH) microbiology laboratory between January 1, 2002 and December 31, 2016 were selected for antimicrobial susceptibility testing, totaling 242 isolates. During this period a total of 9,162 fecal samples from cattle had been submitted for *Salmonella* culture, rendering a prevalence of 2.64% for *Salmonella* culture positive. Some of the isolates were recovered from dairy cattle exhibiting clinical signs of salmonellosis, and the remaining isolates were recovered through the VMTH Infectious Disease Control (IDC) program from asymptomatic dairy cattle. Relevant variables such as year and month of sample collection, location (county) of farm of origin, reason for sample collection, rough age group of each animal sampled, sex of animal, serotype, serogroup, and results of any former antimicrobial susceptibility tests were retrieved from the veterinary hospital records database for each *Salmonella* isolate, assuring client confidentiality.

### Microbiologic procedure for *Salmonella* detection

*Salmonella* were isolated from submitted fecal samples using standardized bacteriologic culture methods, including selective enrichment in selenite broth (Vet Med Biological Media Services, Davis, CA) overnight with subculture of selenite broth to xylose lysine tergitol 4 (Hardy Diagnostics, Santa Maria, CA) and Hektoen enteric (Hardy Diagnostics) agars[10]. As a standard, approximately 10 grams or 10 ml of liquid feces were used for the enrichment in 100 ml selenite broth. Less commonly, when minimal sample was submitted to the laboratory (e.g. rectal swabs), approximately 0.5–1 grams of feces were used to inoculate 10 ml of selenite broth. Confirmation of suspect colonies was performed using biochemical testing and/or matrix-assisted laser desorption-ionization mass spectrometry (MALDI-TOF; Bruker Daltonics, Fremont, CA). Confirmed *Salmonella* isolates were sent to the National Veterinary Services Laboratories in Ames, Iowa for serotyping using standard protocols. Isolates were frozen as stabilates at -80°C until susceptibility testing and were revived on 5% sheep blood agar (Hardy Diagnostics, Santa Maria, CA) incubated in 5% CO_2_ at 35°C. Isolates were not passaged further before antimicrobial susceptibility testing. Throughout the study period, every tenth isolate tested for antimicrobial susceptibility was also re-confirmed as *Salmonella* using MALDI-TOF.

### Antimicrobial susceptibility testing

Antimicrobial susceptibility testing was conducted using a microbroth dilution (MBD) method per Clinical Laboratory Standards Institute (CLSI) guidelines [11]. All isolates were tested against a standardized National Antimicrobial Resistance Monitoring System (NARMS) panel (Thermo Fisher, Sensititre) for aerobic Gram negative bacteria that included penicillins (ampicillin), beta-lactam/beta-lactamase inhibitor combinations (amoxicillin/clavulanic acid), cephalosporins (ceftriaxone, ceftiofur, and cefoxitin), quinolones (ciprofloxacin and nalidixic acid), phenicols (chloramphenicol), sulfas (sulfisoxazole and sulfamethoxazole/trimethoprim), tetracyclines (tetracycline), macrolides (azithromycin), and aminoglycosides (gentamicin, and streptomycin) [12]. Some isolates had already been tested for antimicrobial susceptibility to some of these agents, but all isolates were retested against this standard panel of 14 antibiotics to assure consistency and improve surveillance of AMR. Positive and negative controls on every MBD plate provided quality assurance. The Sensititre (Trek Diagnostic Systems, Oakwood Village, OH, USA) gram negative NARMS plate (CMV3AGNF) was used for testing isolates. Plates were read using the Sensititre Vizion System^®^ (Thermo Fisher) and minimum inhibitory concentrations (MIC) were interpreted using NARMS breakpoints [13]. Weekly quality control was conducted using five strains of bacteria: *Escherichia coli* 25922, *Escherichia coli* 35218, *Enterococcus faecalis* 29212, *Staphylococcus aureus* 29213, and *Pseudomonas aeruginosa* 27853. All MIC results for these strains were interpreted using ranges recommended by CLSI for quality control [11].

### Data analysis

The statistical software programs Excel (Microsoft Corp., Redmond, WA), JMP and SAS (SAS Institute Inc., Cary, NC) were used to process data for the *Salmonella* isolates and analyze the serotype and AMR patterns of isolates for associations with relevant variables.

Using the FREQ function in SAS, the Cochran-Armitage trend test was used to evaluate temporal trends in the prevalence of *Salmonella* resistant to drug being tested for isolates obtained from 2002 to 2016. Cochran-Armitage Trend test was also used to evaluate shifts in serotype distribution for the top 7 serovars (each representing at least 4% or more of the total *Salmonella* isolates in the study), as well as one group containing all the remaining serotypes. Serovar in the “others” group represent individual serotypes representing 2% or less of the all *Salmonella* isolates used in the study. This was done because serotype was considered an important potential confounding variable. Furthermore, temporal trends in the prevalence to individual antimicrobial drugs tested to evaluate the role of serotype on antimicrobial resistance trends. This was conducted using the LOGISTIC function in SAS, where the dependent variable was the binary variable determining resistance or not to an antimicrobial, and the independent variables were the top six serotypes, year interval (2002–2009 vs 2010–2016) when isolate was collected, and the interaction of these two variables.

Multiple logistic regression models using GLIMMIX function in SAS were used to evaluate the effect of sex, age, serotype and gender on the probability of *Salmonella* being resistant to each antimicrobial drug tested, as well as multidrug resistant (MDR). MDR was defined as resistance to at least one agent in three or more antimicrobial categories [14]. In these logistic regression models, the binary dependent variable determined if isolates were or not MDR. Year interval (2002–2009 vs 2010–2016) when isolates were collected was included in all models as an independent variable to evaluate temporal changes on antimicrobial resistance. These two year periods were selected to compare the two halves of time period evaluated, as well as due to legislation related to antimicrobial use that occurred after 2009, such as change on how ceftiofur could be used in an extra-label matter in livestock [15]. A cluster was created within serotype with serotypes for which less than 10 isolates were available. For all models, a *P*-value of < 0.05 was considered to be a significant difference.

A heat map was generated using in JMP to display the distribution of *Salmonella* nonsusceptible to ceftriaxone by serotype. Isolates were labeled as nonsusceptibility to ceftriaxone if classified as either intermediate or resistant to ceftriaxone.

## Results

### Antimicrobial susceptibility profiles

**Fig 1:**
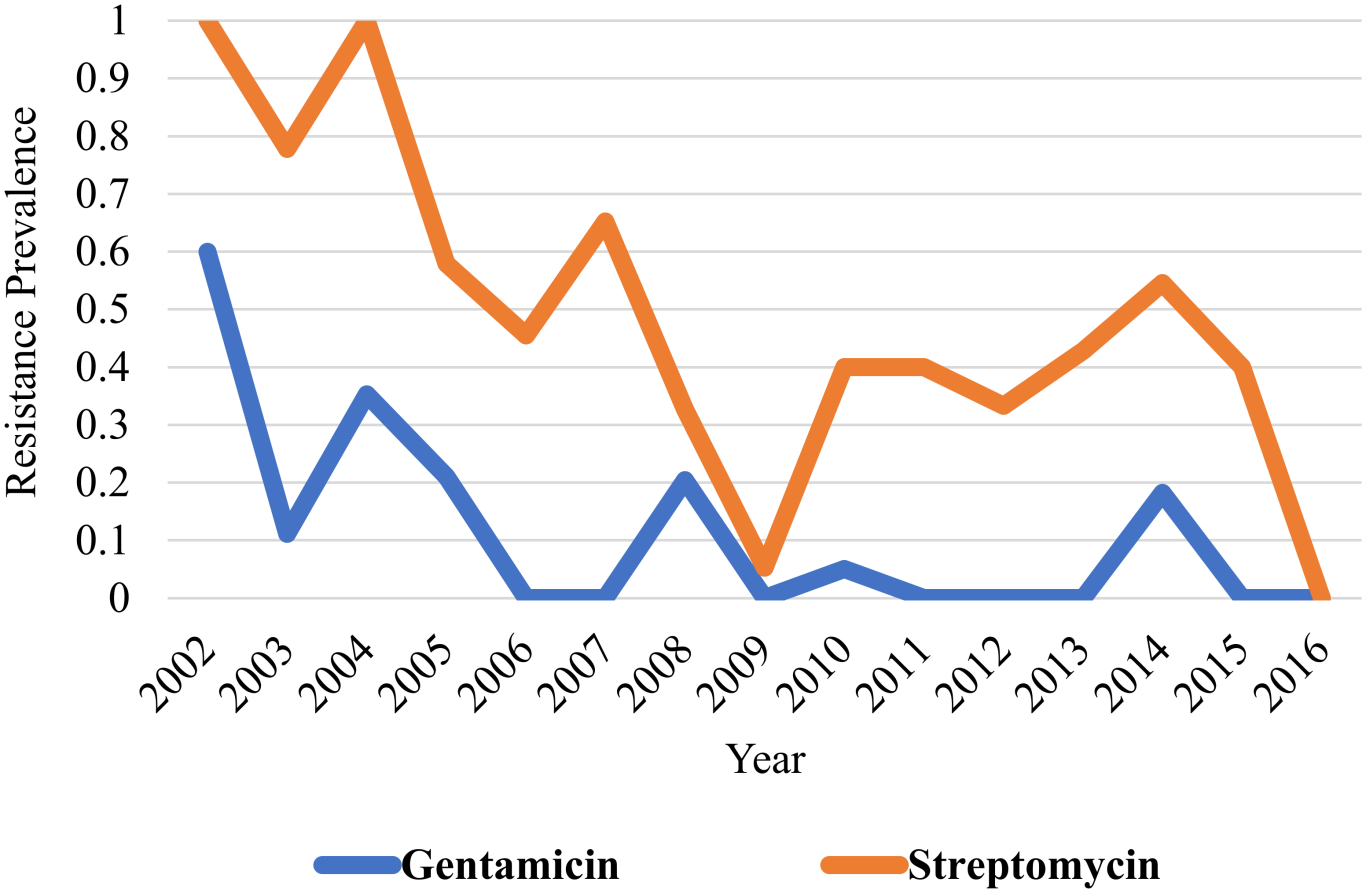
Prevalence of antimicrobial resistance to gentamicin and streptomycin antimicrobials exhibits a decreasing linear trend from 2002–2016. There are significantly higher odds for resistance to aminoglycosides for the 2002–2009 period when compared to the 2010–2016 period (OR: 2.03, 95% CI: 1.1–3.7).

**Fig 2:**
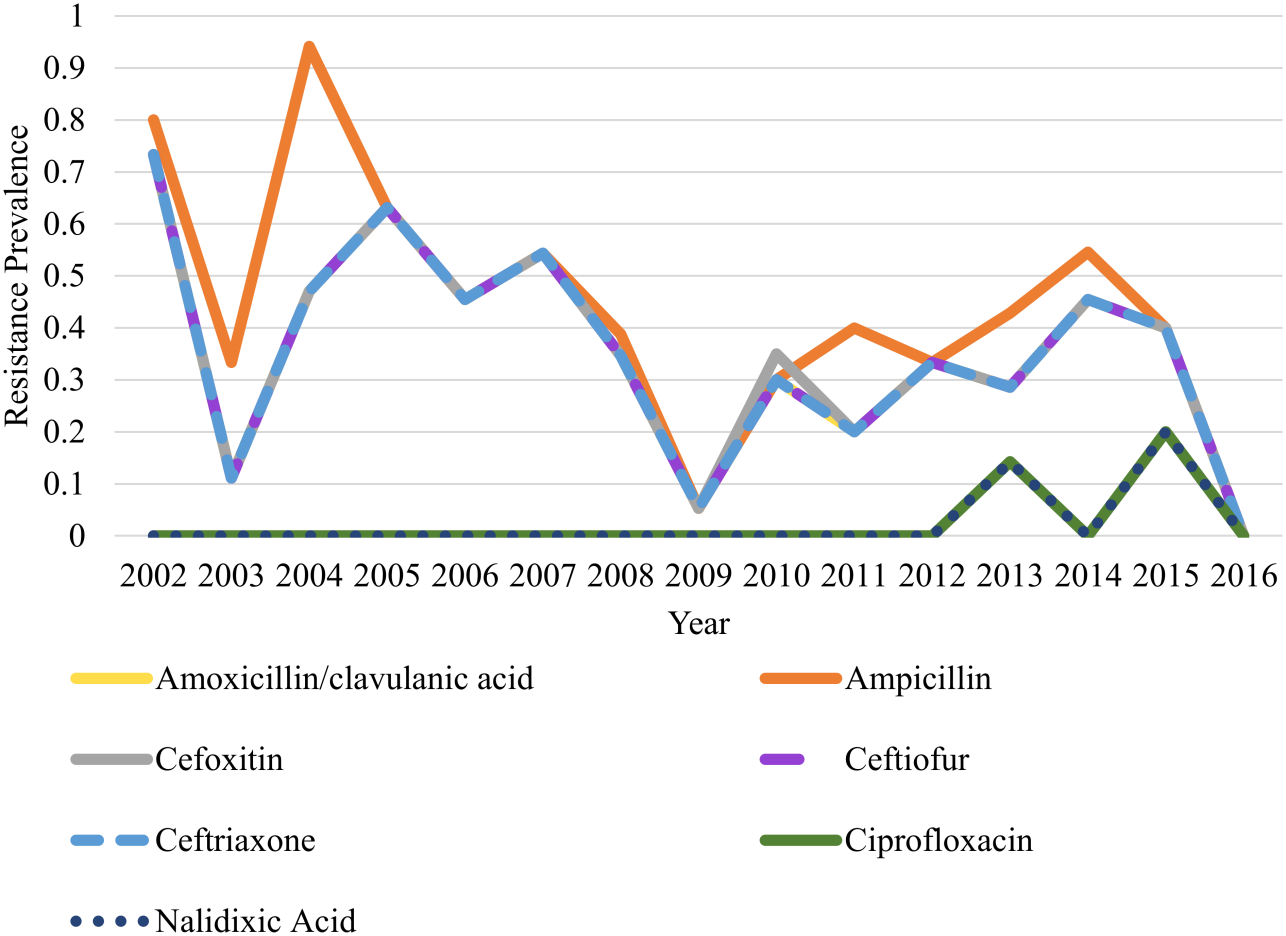
Prevalence of antimicrobial resistance to nalidixic acid has an increasing trend from 2002–2016, and antimicrobial resistance to beta-lactam antimicrobials and a beta-lactam/beta-lactamase inhibitor combination exhibit decreasing linear trends from 2002–2016. There are significantly higher odds for resistance to amoxicillin/clavulanic acid (OR: 1.79, *95% CI*: *0*.*94–3*.*4)* and ampicillin (OR: 1.87, *95% CI*: *1*.*007–3*.*5*) for the 2002–2009 period when compared to the 2010–2016 period. There was no significant difference in odds for resistance to cephalosporins for the 2002–2009 period when compared to the 2010–2016 period (OR: 1.6, *95% CI*: *0*.*87–3*.*1*). Resistance to nalidixic acid occured only in the 2010–2016 period.

**Fig 3:**
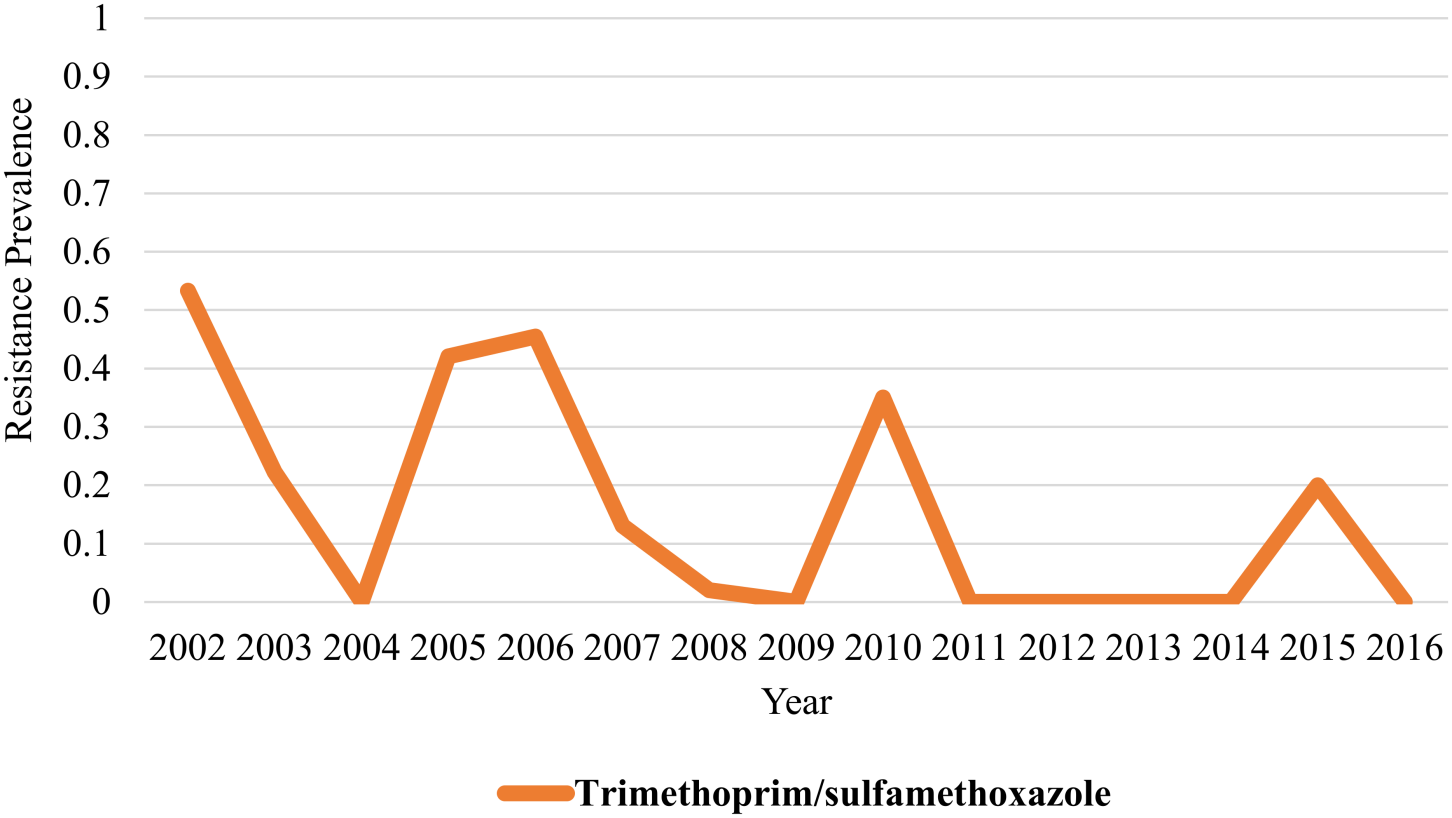
Prevalence of antimicrobial resistance to folate pathway inhibitors exhibits a decreasing linear trend from 2002–2016. However, no significantly higher odds for resistance to folate pathway inhibitors for the 2002–2009 period when compared to the 2010–2016 period (OR: 1.18, *95% CI*: *0*.*50–2*.*76*).

**Fig 4:**
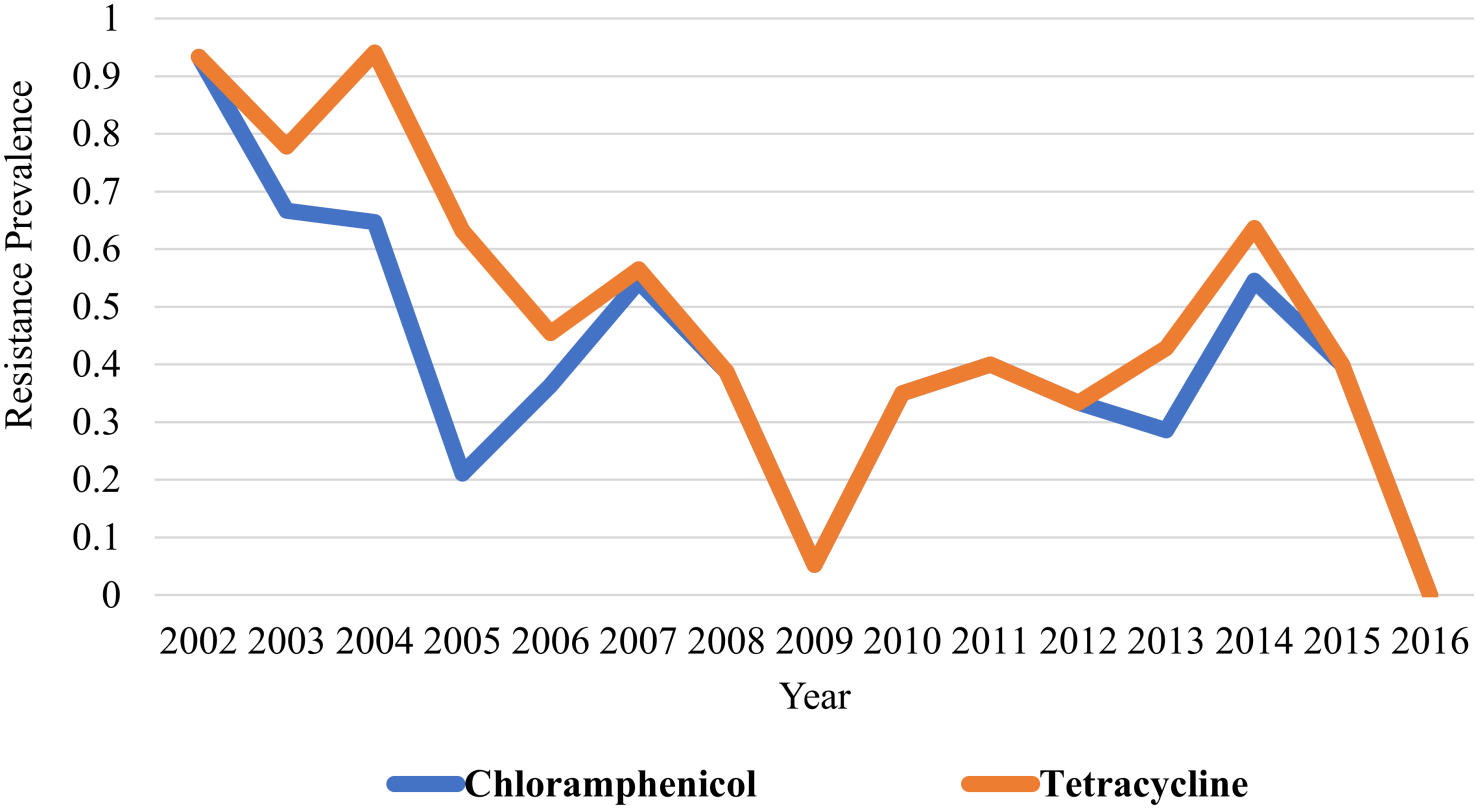
Prevalence of antimicrobial resistance to tetracycline and chloramphenicol exhibit decreasing linear trends from 2002–2016. There are significantly higher odds for resistance to tetracycline for the 2002–2009 period when compared to the 2010–2016 period (OR: 1.87, *95% CI*: *1*.*017–3*.*4*). No significant difference in odds for resistance to phenicols for the 2002–2009 period when compared to the 2010–2016 period (OR: 1.6, 95% CI: 0.87–3.1).

Cochran-Armitage trend test revealed a significant trend for reduction in the annual prevalence of antimicrobial resistance for *Salmonella* obtained from 2002–2016 for amoxicillin (*P value* 0.001), ampicillin (*P value* <0.001), cefoxitin (*P value* 0.001), ceftiofur (*P value* 0.001), ceftriaxone (*P value* 0.001), chloramphenicol (*P value* 0.001), gentamicin (*P value* <0.001), streptomycin (*P value* <0.001), tetracycline (*P value* <0.001), and trimethoprim/ sulfamethoxazole (*P value* 0.002)(Figs 1 to 4). The only antimicrobial with an increasing trend in the annual prevalence of antimicrobial resistance for *Salmonella* from 2002–2016 was the quinolone drug nalidixic acid (*P value* 0.004). Azithromycin, sulfisoxazole and ciprofloxacin were not included in this analysis because no isolates in this study was resistant to these drugs.

Aminoglycosides (gentamicin, streptomycin; Fig 1)(OR: 2.03, *95% CI*: *1*.*1–3*.*7*, *P value 0*.*02*), penicillins (ampicillin; Fig 2)(OR: 1.87, *95% CI*: *1*.*007–3*.*5*, *P value 0*.*04*), and tetracycline (Fig 4)(OR: 1.87, *95% CI*: *1*.*017–3*.*4*, *P value 0*.*04*) showed significantly greater odds for antimicrobial resistance in the 2002–2009 period when compared to the 2010–2016 period. No significant difference in the odds for resistance to cephalosporins (OR: 1.6, *95% CI*: *0*.*87–3*.*1*, *P value 0*.*12*), folate pathway inhibitors (trimethoprim/ sulfamethoxazole; Fig 3)(OR: 1.18, *95% CI*: *0*.*50–2*.*76*, *P value 0*.*69*), beta-lactam/beta-lactamase inhibitor combinations (amoxicillin/clavulanic acid; Fig 2)(OR: 1.79, *95% CI*: *0*.*94–3*.*4*, *P value 0*.*07*), and phenicols (OR: 1.6, *95% CI*: *0*.*87–3*.*1*, *P value 0*.*12*) was observed when the 2002–2009 period was compared to the 2010–2016 period (Figs 1 to 4).

Minimum inhibitory concentration distribution for *Salmonella* for each antimicrobial tested is displayed in Table 1. Decreased susceptibility to ciprofloxacin (DSC), defined as isolates with an MIC > = 0.12 μg/ml, is used as a marker for emerging fluoroquinolone resistance [16]. Although no isolate was classified as resistant to ciprofloxacin, two percent of isolates fell within the DSC category (Table 1). These were the same two isolates classified as resistant to nalidixic acid.

**Table 1:** Percent distribution of MIC resistant for *Salmonella* isolates (n = 242) for isolates. Highlighted areas in blue correspond to susceptible, in yellow correspond to intermediate, and in red highlighted area correspond to resistant.

	% Distribution of MICs (μg/ml)														
Antimicrobial	0.015	0.03	0.06	0.12	0.25	0.5	1	2	4	8	16	32	64	128	256
**AU**							50	3		2	4	40			
**AM**							45	8	1			46			
**AZO**									75	24	1				
**FOX**								21	25	13		40			
**XNL**					1	3	52	2	1	40					
**CRO**					58	2				2	16	16	5		
**CHO**									18	38	1	43			
**CIP**	46	50	2		1	1									
**GM**					16	58	10	2	1		13				
**NA**							1	15	78	6		1			
**STR**									6	31	11	10	41		
**FIS**											1	1	2	13	83
**TE**									50			50			
**SXT**				49	19	13	2	1	16						

AU, amoxicillin/clavulanic acid; AM, ampicillin; AZO, azithromycin; FOX, cefoxitin; XNL, ceftiofur; CRO, ceftriaxone; CHO, chloramphenicol; CIP, ciprofloxacin; GM, gentamicin; NA, nalidixic acid; STR, streptomycin; FIS, sulfisoxazole; TE, tetracycline; SXT, trimethoprim/sulfamethoxazole.

Of the nine antimicrobial classes represented on the standardized NARMS panel of drugs, all isolates were susceptible to azithromycin (macrolide), with two isolates nonsusceptible to ciprofloxacin. Resistance to the quinolone nalidixic acid was observed in 0.8% (2/242) of *Salmonella* isolates. These two isolates were classified as resistant to nalidixic acid and included in both the nonsusceptibility prevalence graph as well as the MDR analyses (Fig 2; Table 2).

**Table 2:** Distribution of pansusceptible and resistant patterns of 242 *Salmonella* isolates to the NARMS gram-negative antimicrobial panel.

Susceptibility Pattern	Count	Prevalence
Pansusceptible	112	46.3%
AuAmFoxXnlCroChoStrTe	38	15.7%
AuAmFoxXnlCroChoStrTeSxt	17	7.0%
AuAmFoxXnlCroChoGmStrTe	16	6.6%
AuAFoxXnlCroStrTeSxt	10	4.1%
AmStrTe	8	3.3%
ChoStrTe	8	3.3%
AuAmFoxXnlCroChoGmStrTeSxt	7	2.9%
Str	7	2.9%
AmChoStrTe	6	2.5%
AuAmFoxXnlCroChoGmTe	6	2.5%
AmChoGmStrTeSxt	2	0.8%
AuAmFoxXnlCroChoNaStrTe	1	0.4%
AuAmFoxXnlCroChoNaStrTeT/S	1	0.4%
AuAmFoxXnlCroStr	1	0.4%
ChoGmStrTe	1	0.4%
FoxChoGmStrTeSxt	1	0.4%

Au, amoxicillin/clavulanic acid; Am, ampicillin; Fox, cefoxitin; Xnl, ceftiofur; Cro, ceftriaxone; Cho, chloramphenicol; Gm, gentamicin; Na, nalidixic acid; Str, streptomycin; Te, tetracycline; Sxt, trimethoprim/sulfimethoxazole

**Fig 5:**
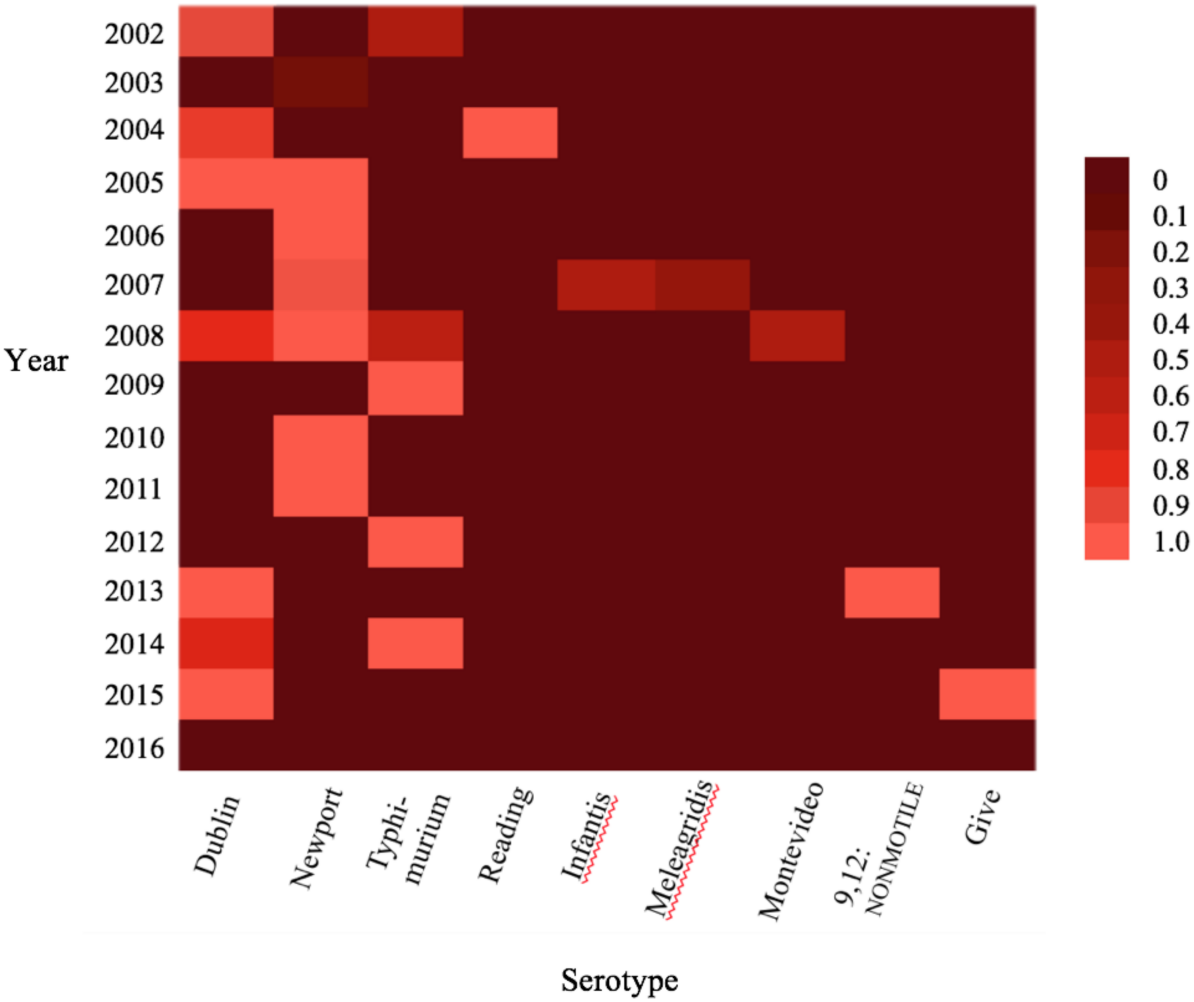
*Salmonella* serotypes Dublin, Newport, and Typhimurium display nonsusceptibility to ceftriaxone in multiple years of the study period. Only serotypes with at least one isolate nonsusceptible to ceftriaxone were included in the analysis (n = 151). The heatmap legend represents the prevalence of ceftriaxone resistant *Salmonella*.

Resistance to ceftriaxone was observed in 40.1% (97/242) of *Salmonella* isolates and was observed in 22.5% (9/40) of the serotypes identified throughout the study period. At least two *Salmonella* isolates per year from 2002–2015 were resistant to ceftriaxone, with susceptibility to ceftriaxone observed among the six *Salmonella* isolates obtained from dairy cattle fecal samples in 2016 (Fig 5). Only serotypes with at least one isolate nonsusceptible to ceftriaxone were included in Fig 5 (n = 151). Because antimicrobial susceptibility testing to ceftiofur and ceftriaxone was the same for all isolate, Fig 5 can also be interpreted as serotypes with at least one isolate nonsusceptible to ceftiofur.

### Serotypes

**Fig 6:**
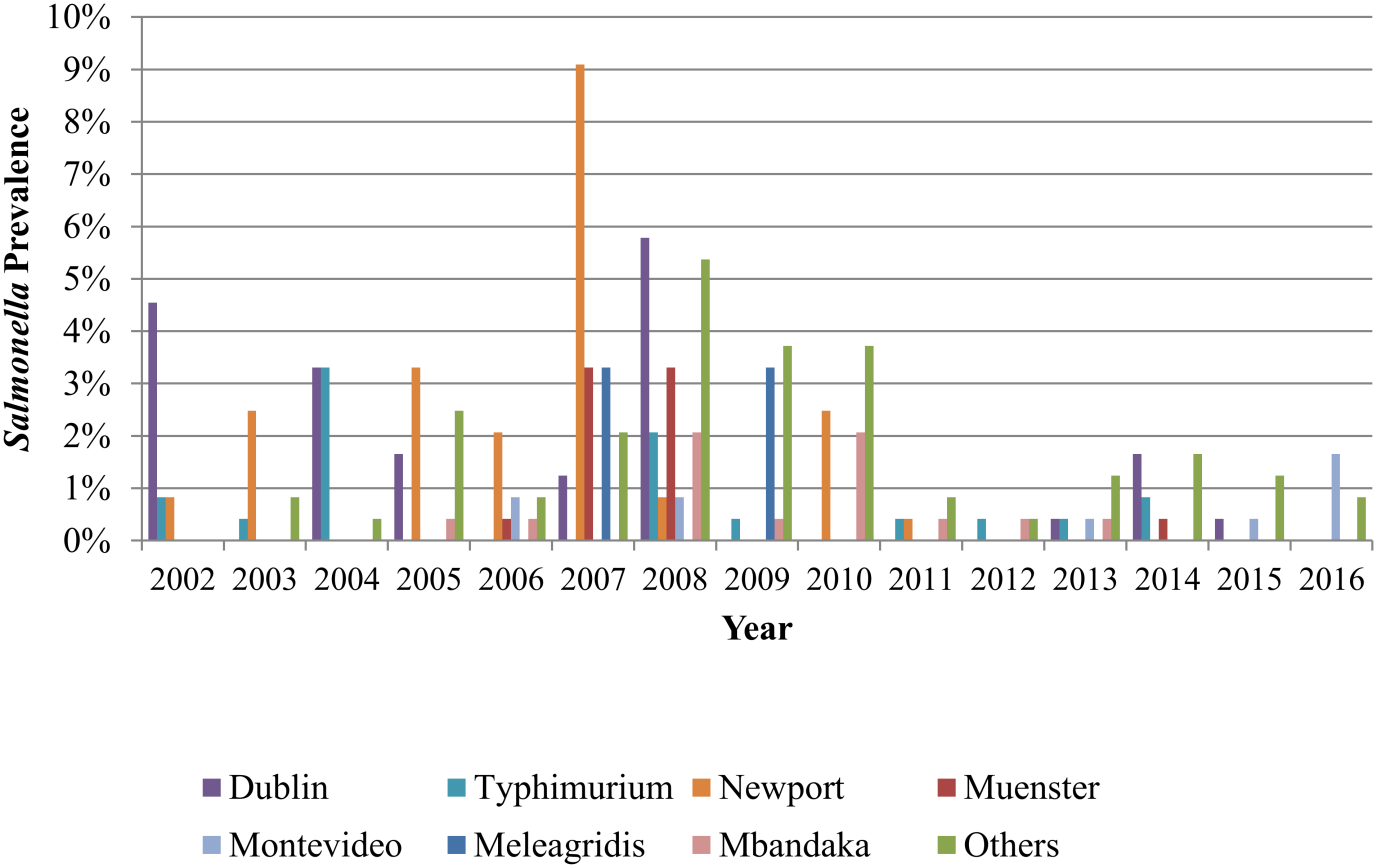
Annual prevalence of *Salmonella* by the top 7 serotypes. “Others” represents the clustering of all other serotypes not included in the top seven.

Cochran-Armitage trend test revealed a significant trend for decreased annual prevalence of serotypes Dublin (*P value* 0.006) and Newport (*P value* 0.005) from 2002–2016. A significant trend for increase was observed for Montevideo (*P value* <0.001), Mbandaka (*P value* 0.044) and others (*P value* <0.001). No significant change towards increasing or decreasing prevalence was observed for Typhimurium (*P value* <0.10), Muenster (*P value* 0.5) and Meleagridis (*P value* 0.038)(Fig 6).

For all antimicrobial tested in the study, prevalence of resistance for the top six serovars was not significantly different for the 2002–2009 period when compared to the 2010–2016 period.

### Multi-drug resistance and serotypes

**Fig 7:**
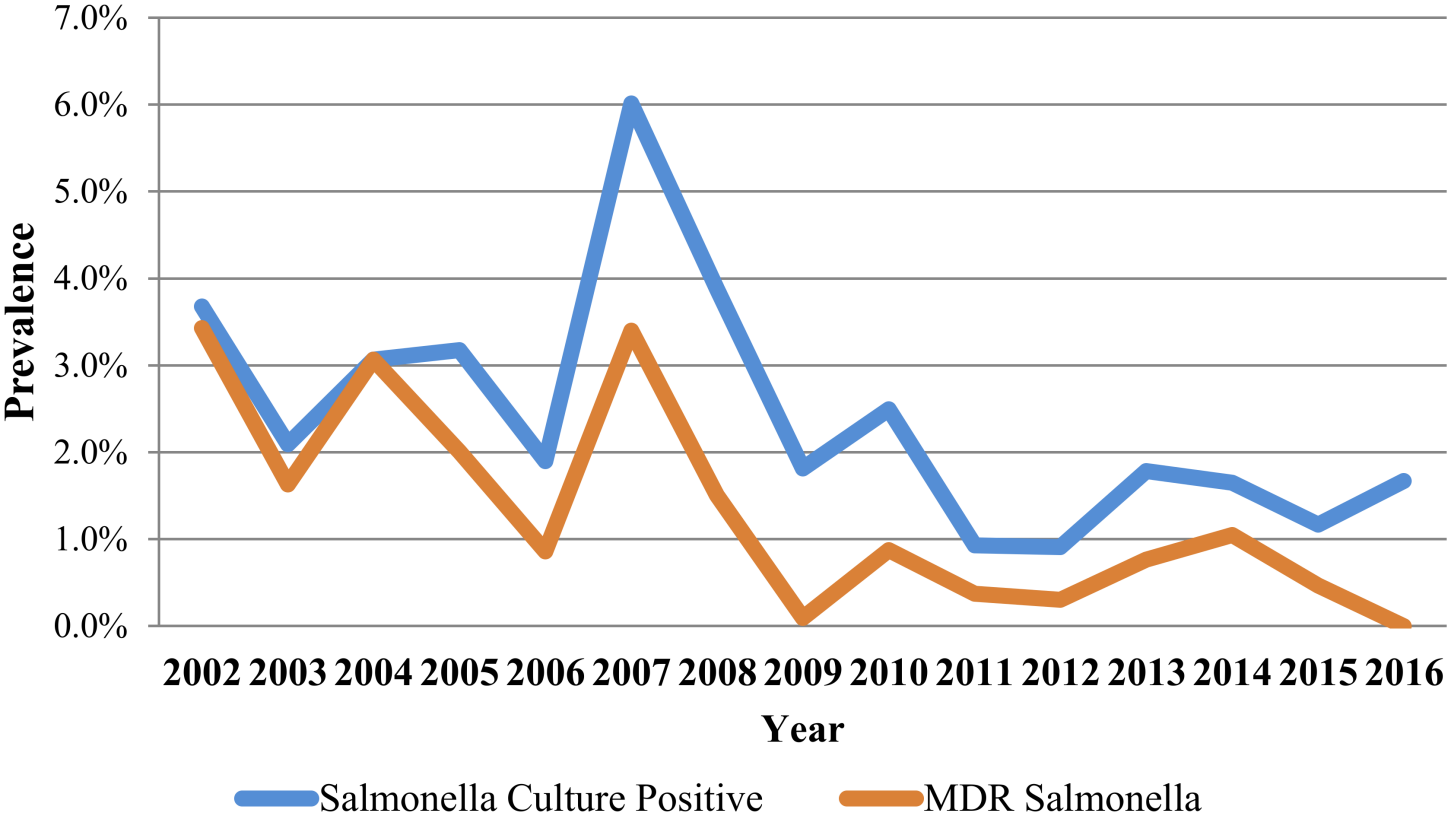
Prevalence of *Salmonella* culture positive and multidrug resistant (MDR) *Salmonella* isolates from fecal samples submitted between 2002 and 2016.

Multi-drug resistance was observed in 50.8% (123/242) of *Salmonella* isolates (Fig 7). The most common MDR pattern was amoxicillin/clavulanic acid—ampicillin—cefoxitin—ceftiofur—ceftriaxone—chloramphenicol—streptomycin—tetracycline (Au-Am-Fox-Xnl-Cro-Cho-Str-Te; 15.7%), and the second and third most common MDR patterns were similar, with the addition of trimethoprim-sulfamethoxazole (Sxt; 7.0%) and gentamicin (Gm; 6.6%) to this base pattern, respectively (Table 2). Approximately 46% of isolates were pansusceptible. Of the 40 unique *Salmonella* serotypes observed over the study period, the most common were Newport (21.5%), Dublin (19.0%), and Typhimurium (9.1%) (Table 3). A significantly higher (*P* value < 0.0001) prevalence of *S*. Dublin was observed in isolates from calves (43%) when compared to isolates from adult animals (10%).

**Table 3:** Distribution of serotypes and percent of multidrug resistance isolates observed among 242 *Salmonella* isolates by time frame.

Serotype	2002 to 2009 (n = 185)		2010 to 2016 (n = 57)	
	% (n)	% MDR (n)*	% (n)	% MDR (n)*
*S*.NEWPORT	24% (45)	100% (45)	12% (7)	100% (7)
*S*.DUBLIN	22% (40)	85% (33)	11% (6)	100% (6)
*S*.TYPHIMURIUM	8% (15)	100% (15)	9% (5)	100% (5)
*S*.MELEAGRIDIS	9% (16)	19% (3)	0% (0)	
*S*.MUENSTER	9% (16)		0% (0)	
*S*.MBANDAKA	4% (8)		14% (8)	
*S*.MONTEVIDEO	2% (4)	25% (1)	11% (6)	
*S*.UGANDA	1% (2)		5% (3)	
*S*. SP. 4,12:i:-	0% (0)		4% (2)	
*S*.BERTA	0% (0)		4% (2)	
*S*.IDIKAN	0% (0)		4% (2)	
*S*.BARRANQUILLA	2% (3)		2% (1)	
*S*. SP. 4,5,12:i:-	1% (2)		2% (1)	50% (1)
*S*.TENNESSEE	1% (2)		2% (1)	
*S*.ORANIENBURG	1% (2)		2% (1)	
*S*.SAINTPAUL	1% (2)		2% (1)	
*S*. rough O:GMS:-	0% (0)		2% (1)	
*S*. sp. (3,12:l,z13:-)	0% (0)		2% (1)	
*S*. sp. (4,5,12:I:)	0% (0)		2% (1)	
*S*. sp. (9,12:NONMOTILE)	0% (0)		2% (1)	100% (1)
*S*.ARIZONAE	0% (0)		2% (1)	
*S*.BRAENDERUP	0% (0)		2% (1)	
*S*.DERBY	0% (0)		2% (1)	
*S*.ENTERIDITIS	0% (0)		2% (1)	
*S*.GIVE	0% (0)		2% (1)	100% (1)
*S*.MUEC	0% (0)		2% (1)	
*S*.THOMPSON	0% (0)		2% (1)	
*S*.SENFTENBERG	3% (5)		0% (0)	
*S*.ALTONA	2% (4)		0% (0)	
*S*.ANATUM	2% (3)		0% (0)	
*S*.HEIDELBERG	2% (3)		0% (0)	
*S*. SP. 3,1:e,h:-	1% (2)		0% (0)	
*S*.HAVANA	1% (2)		0% (0)	
*S*.INFANTIS	1% (2)	100% (1)	0% (0)	
*S*. SP. 3,12:NONMOTILE	1% (1)		0% (0)	
*S*.AGONA	1% (1)		0% (0)	
*S*.CERRO	1% (1)		0% (0)	
*S*.LEXINGTON	1% (1)		0% (0)	
*S*.MUENCHEN	1% (1)		0% (0)	
*S*.POONA	1% (1)		0% (0)	
*S*.READING	1% (1)	100% (1)	0% (0)	

* Percent of isolates for that serotype classified as multidrug resistant.

### Risk factors

Prevalence of multidrug resistance in *Salmonella* isolates significantly varied based on age group of animal, serotype, and submission type of *Salmonella* isolates. There was a significantly higher odds ratio of isolating MDR *Salmonella* from calves when compared to adult cattle (*P-*value *=* 0.0004) and from disease suspects when compared to samples collected due to the hospital IDC program (*P-*value *=* 0.001) (Table 4). Location (county) of farm of origin (*P*-value = 0.79) and sex of animal (*P*-value = 0.63) were not significantly associated with a higher prevalence of *MDR Salmonella*. Year group (2002–2009 vs 2010–2016) did not have a significant effect on the odds ratio of isolating MDR *Salmonella* (*P-*value *=* 0.20). *Salmonella* serotype had a significant effect on the prevalence of MDR isolated (*P-*value *<* 0.0001). If an isolate was serotype Newport, Meleagridis, Typhimurium or Dublin a significantly higher probability for MDR was observed when compared to a reference group composed of serotypes for which less than 10 isolates were available. All other risk factors evaluated were not significantly associated with a higher probability of isolating MDR *Salmonella*.

**Table 4:** Evaluation of risk factors for prevalence of multidrug resistant (MDR) *Salmonella* from 2002 to 2016 (n = 242), based on age, sample submission type, serotype and year group isolated.

Factor	% MDR Prevalence^1^(total count)	OR (95% C.I.)^2^	*P*-value
**Age Group**			0.0001
Adult	39% (170)	22.0 (3.9–125.7)	
Calves	82% (67)	Reference	
**Submission Type**			0.0004
Suspects	76% (107)	13.7 (2.8–66.8)	
IDC	31% (135)	Reference	
**Serotype**			<0.0001
Dublin	85% (46)	112.8 (11.8–1,072)	
Mbandaka	0% (16)	-**	
Meleagridis	19% (16)	11.8 (1.1–121.1)	
Montevideo	10% (10)	0.14 (0.009–2.0) ***	
Muenster	0% (18)	- **	
Newport	100% (52)	-**	
Typhimurium	100% (22)	-**	
Other*	10% (62)	Reference	

^1^ Prevalence of multidrug resistant *Salmonella* within each variable and number of isolates within each category.

^2^ Odds ratio for having *Salmonella* being MDR due to the factor being evaluated. The 95% confidence interval is in parentheses.

* Cluster of serotypes for which less than 10 isolates were available.

** Odds ratio not calculated because either all isolates in that category were multidrug resistant or all isolates in that category were not multidrug resistant.

*** 95% CI for the odds ratio included 1, indicating lack of evidence for isolated being MDR

## Discussion

Identifying trends in AMR in dairy cattle *Salmonella* isolates provides vital surveillance data to the scientific and medical communities to guide research, judicious antimicrobial use, and selection of effective treatment plans. The decreasing linear trends in antimicrobial resistance prevalence observed for three antimicrobial classes from 2002–2016 support the notion that susceptibility profiles of *Salmonella* isolated from northern California dairy cattle fecal samples are changing over time (Figs 1–4). A study conducted in bovine *Salmonella* enterica submitted to the Wisconsin Veterinary Diagnostic Laboratory from 2006–2015 observed similar findings, with trend for decreasing antimicrobial resistance observed overtime for gentamicin, neomycin, and trimethoprim sulfamethoxazole [17]. While a decreasing trend in resistance patterns for most antimicrobial classes was observed, there is still a problem with antimicrobial resistance for cephalosporins and quinolone drugs as evidenced by lack of significant difference in odds for resistance to cephalosporins between the 2002–2009 and 2010–2016 periods and identification of isolates resistance to quinolones in 2013 and 2015.

The fact that there was no significant difference in odds ratio for resistance to cephalosporins for the 2002–2009 period when compared to the 2010–2016 period is worth noting because cephalosporins are key drugs for treating human patients with severe nontyphoidal *Salmonella* infections, especially ceftriaxone. While ciprofloxacin is commonly prescribed to adults with salmonellosis, third-generation cephalosporins such as ceftriaxone are the preferred treatment for children [18]. The presence of nonsusceptibility to ceftriaxone in multiple serotypes known to cause foodborne illness in humans such as *S*. Dublin, *S*. *Newport* and *S*. *Typhimurium* show that continued surveillance of dairy cattle *Salmonella* isolates is warranted (Fig 5)[19].

Approximately 50% of *Salmonella* isolates in our study were MDR, this is within the expected prevalence for MDR in cattle, with a study on antimicrobial resistance among *Salmonella* from dairy cattle in the Northeastern US from 2004–2011 reporting a prevalence of MDR *Salmonella* of 46% [20]. When evaluating risk factors for prevalence of MDR *Salmonella*, age group, and submission type played a significant role (Table 4). It was not unexpected to observe that *Salmonella* from calves had a higher probability of being MDR when compared to adult animals: this has been previously observed that calves have a higher proportion of MDR enteric bacteria when compared to cows [21, 22]. One hypothesis is that undeveloped intestinal microflora in young calves could influence higher colonization of younger calves by pathogenic and antimicrobial resistant enteric bacteria. Resistance to colonization by bacteria with a higher fitness cost, such as antimicrobial-resistant bacteria and pathogenic enteric bacteria, may follow as the calves’ indigenous microflora matures and the enteric microbiota diversity increases, resulting in a decreased prevalence of resistant bacteria [23, 24]. Additionally, a significantly higher (*P* value < 0.0001) prevalence of *S*. Dublin was observed in calves (43%) when compared to adult animals (10%), and due to the association of *S*. Dublin with being MDR, the higher prevalence of infection of calves with this serotype likely affected the higher probability of calves being isolated with MDR *Salmonella*.

In our study we also observed a higher prevalence of MDR *Salmonella* in animals that were sampled based on being suspects for having clinical salmonellosis versus as part of the standard veterinary hospital IDC protocol. A hypothesis for this finding may be that animals that are suspect for salmonellosis may have higher odds for being recently treated with one or more antimicrobials due to clinical signs associated with salmonellosis prior to arriving at the hospital or during hospitalization, and that selection pressure for antimicrobial resistance may have occurred. This could occur either as a consequence of MDR *Salmonella* to survive exposure to multiple antimicrobial treatments with difference drug classes, as well as the selective pressure antimicrobial treatments exert on enteric microbiota, facilitating the dominance of a MDR isolate that otherwise would not been able to compete with commensal microbiota, facilitating therefore the clonal dissemination of a MDR isolate [25]. One study observed that recent treatment of adult cattle with antimicrobials significantly increased the risk of shedding *Salmonella* group B (OR = 2.1; 95% CI: 1.4–3.0) [26]. Clinical signs of bovine salmonellosis may include fever, diarrhea, anorexia, dehydration, decreased milk production, abortion, and endotoxemia, although many infections remain subclinical [27]. In comparison to animals that may be at the veterinary hospital for reasons unrelated to those expected for an animal with salmonellosis, they may not have recently been exposed to the same antimicrobial selection pressures that would increase the odds for isolating multidrug resistant *Salmonella*. In agreement with our hypothesis, a previous study observed that multidrug resistance was found to be highly prevalent among isolates from cattle with clinical signs of salmonellosis [28]. As an example, *S*. Cerro is frequently isolated from cattle subclinical for salmonellosis, and *S*. Cerro isolates are usually pansusceptible [29]. Animals shedding *Salmonella* subclinically highlight the importance of IDC protocols, which is of higher relevance in large animal veterinary hospitals which have patients that may have a depressed immune system and may be more vulnerable to developing clinical salmonellosis after exposure [30].

Although no isolate was classified as resistant to ciprofloxacin, two percent of isolates fell within the DSC category (Table 1). Quinolone antibiotics act by inhibiting the topoisomerase enzymes, DNA gyrase and topoisomerase IV, which maintain the level of supercoiling in the bacterial DNA[31]. Quinolone resistance can result from point mutations in housekeeping genes (e.g., *gyrA*), rather than the presence of resistance genes, even though the presence of some resistance genes (e.g., *qnr* gene) may confer low level resistance and could result in reduced susceptibility without changing the classification of an isolate from susceptible to intermediate or resistant [32, 33]. The identification of these DSC isolates in 2013 and 2015 highlights the importance of continued monitoring of potential increased selection of resistance to fluoroquinolone drugs in livestock and evaluation of potential spread through direct contact or the food chain.

The 2015 NARMS report outlined an increase in MDR prevalence of *Salmonella* isolated from humans from ~9.5% from 2009–2014 to 12% in 2015 driven by a *S*. Typhimurium variant named 4,[5],12:i:- [19]. In this study, four *Salmonella* isolates were serotype 4,[5],12:i:-, and these were observed in 2009, 2010 (2), and 2014. Multidrug resistance was observed only in the most recent (2014) of the four 4,[5],12:i:- isolates, with its susceptibility pattern nearly matching the frequently observed AST pattern as outlined by the NARMS report (Table 2). Additionally, the NARMS report raised concerns about MDR in *S*. Dublin. Although this serotype is host adapted to cattle, it is becoming more prevalent in humans and tends to cause severe infection. The NARMS report also stated that MDR in *Salmonella* Dublin continues to increase, being responsible for 11 out of 12 human *Salmonella* isolates and 28 out of 31 cattle isolates being reported [34]. Of the 46 *S*. Dublin isolates analyzed in this study, 85% were MDR, with 100% of *S*. Dublin isolates in the more recent 2010–2016 period displaying MDR (Table 3). A recent study that conducted whole genome sequencing of *Salmonella* isolated from humans and cattle between 2008 and 2012 from NY and WA, observed a close similarity between *S*. Dublin resistance genes as well as plasmids between *Salmonella* isolated from humans and cattle. This held true between different years, with geographical location having a greater influence on the differences observed, resulting in the presence of *aadB* and *cmlA*, as well as streptomycin resistance genes in isolates from WA but not NY [35]. This highlights that cattle continue to be an important potential reservoir of *S*. Dublin to humans, and selection of antibiotic resistance in cattle could increase the risk of multidrug resistance among *Salmonella* isolates from humans.

The three most common serotypes observed in the 2002–2009 period of our study (*S*. Newport, *S*. Dublin, and *S*. Typhimurium) were also frequently observed by the United States Department of Agriculture’s Food Safety and Inspection Service (USDA-FSIS) among isolates obtained from ground beef at slaughter houses. Specifically, *S*. Dublin was consistently among the top three serotypes in ground beef reported annually by FSIS during 2007–2009 and is among the top ten reported ground beef *Salmonella* serotypes since 2003 [36]. *Salmonella* is known to be host-adapted in cattle, and it is therefore not unexpected to find it in ground beef [37]. A trend for a lower prevalence of *S*. Dublin and *S*. Newport from 2002–2016 was observed in our study. *S*. Montevideo was also observed less frequently during the 2002–2009 period in our study than in USDA-FSIS reports during the same time frame. *S*. Montevideo was the most prevalent serotype reported annually by USDA-FSIS during 2002–2009, whereas only 2% (4/185) of isolates in our study were *S*. Montevideo from 2002–2009. We also observed a trend from higher prevalence of *S*. Montevideo from 2002–2016. The reason for this difference and trend is not clear.

Of the four most common serotypes observed in the 2010–2016 period in our study (*S*. Mbandaka, *S*. Newport, *S*. Dublin, and *S*. Montevideo), USDA-FSIS similarly reported high prevalence of each except for *S*. Mbandaka throughout 2010–2016. Our study reported a relatively high 14% (8/57) prevalence of *S*. Mbandaka in the 2010–2016 period, as well as an increasing trend for prevalence of *S*. Mbandaka from 2002–2016. Based on USDA-FSIS data, *S*. Mbandaka has historically been of low-to-variable importance as a foodborne pathogen in ground beef, particularly in recent years. However, a study conducted with data on *Salmonella* prevalence in commercial ground beef in the United States from 2005–2007 observed a high prevalence of *S*. Mbandaka from ground beef [38]. Disparities observed between our findings and NARMS or USDA-FSIS reports could be related to the fact that our samples were feces rather than ground beef, and highlights the importance of recognizing the potential bias when using data from NARMS or USDA-FSIS reports to predict prevalence of different serotypes at the farm level. However, efforts have been initiated to evaluate the feasibility of conducting on-farm sampling to monitor AMR [39].

Shift in dominance of specific serotypes has been considered a factor that could have affected temporal changes in the prevalence of antimicrobial resistance *Salmonella* overtime. As already mentioned, we observed trends for decreasing (Dublin and Newport) and increasing (Montevideo and Mbandaka) prevalence from 2002–2016 for some of the top serotypes observed in *Salmonella* from our study. However, no significant temporal shift in prevalence of antimicrobial resistance to any of the drugs tested was found to be associated with temporal changes in serotype prevalence in our study. One study monitoring bovine *Salmonella* isolates from dairy cattle in the northeastern United States isolated from samples submitted to an animal health diagnostic center from 2004–2011, observed a significant increase in the trend for prevalence of resistance to ceftifour (*P* value 0.002), as well as a decreasing trend in prevalence of resistance to spectinomycin (*P* value 0.003) among *S*. Newport isolates [20]. In that study, no trend for increase or decrease in antimicrobial resistance was observed for other serotypes. This furthermore highlights the importance of evaluating temporal trends in antimicrobial resistance that could be caused by shifts in prevalence of specific serotype.

Limitations of this study include sampling bias, as samples collected from animals admitted to the Veterinary Medical Teaching Hospital could be influenced by health status of the animal as well as perceived value of the animal to the owner. Therefore, data from our study does not necessarily represent the prevalence of *Salmonella* or antimicrobial resistance patterns of the cattle population in general in this region of California, but rather that of a large animal veterinary teaching facility.

## Conclusion

The odds of isolating multidrug resistant *Salmonella* from cattle in this veterinary hospital had a decreasing trend over the two time periods studied. Despite reduced prevalence of resistance to three drug classes in the 2010–2016 period as compared to the 2002–2009 period, a lack of a significant reduction in resistance for important drug classes such as cephalosporins, and quinolones highlight the relevance of continual AMR surveillance in cattle with *Salmonella* infections in future interventions. A lower prevalence of MDR isolates was observed for the IDC protocol sampling compared to sampling conducted in suspect clinical salmonellosis cases; however, this does not reduce the importance for IDC protocols to reduce the spread of *Salmonella* in veterinary hospitals from subclinical shedders.
